# Mortality Rates during Cholera Epidemic, Haiti, 2010–2011

**DOI:** 10.3201/eid2203.141970

**Published:** 2016-03

**Authors:** Francisco J. Luquero, Marc Rondy, Jacques Boncy, André Munger, Helmi Mekaoui, Ellen Rymshaw, Anne-Laure Page, Brahima Toure, Marie Amelie Degail, Sarala Nicolas, Francesco Grandesso, Maud Ginsbourger, Jonathan Polonsky, Kathryn P. Alberti, Mego Terzian, David Olson, Klaudia Porten, Iza Ciglenecki

**Affiliations:** Epicentre, Paris, France (F.J. Luquero, M. Rondy, A.-L. Page, B. Toure, M.A. Degail, S. Nicolas, F. Grandesso, M. Ginsbourger, J. Polonsky, K.P. Alberti, K. Porten);; Ministry of Health, Port-au-Prince, Haiti (J. Boncy);; Médecins Sans Frontières, Paris (A. Munger, M. Terzian, D. Olson);; Médecins Sans Frontières, Geneva, Switzerland (H. Mekaoui, E. Rymshaw, I. Ciglenecki)

**Keywords:** cholera, burden, mortality, Haiti, outbreak, epidemic, bacteria

## Abstract

The 2010 cholera epidemic in Haiti was one of the largest cholera epidemics ever recorded. To estimate the magnitude of the death toll during the first wave of the epidemic, we retrospectively conducted surveys at 4 sites in the northern part of Haiti. Overall, 70,903 participants were included; at all sites, the crude mortality rates (19.1–35.4 deaths/1,000 person-years) were higher than the expected baseline mortality rate for Haiti (9 deaths/1,000 person-years). This finding represents an excess of 3,406 deaths (2.9-fold increase) for the 4.4% of the Haiti population covered by these surveys, suggesting a substantially higher cholera mortality rate than previously reported.

## Abstract 2

Actual rates were higher than rates calculated from healthcare facility reports.

On October 22, 2010, the first cholera case in a century was confirmed in Haiti (*1*), one of the poorest countries in Latin America and the Caribbean. The ensuing cholera epidemic progressed rapidly, affecting all departments in the country within 1 month. Haiti’s Ministère de la Santé Publique et de la Population (MSPP) led a large intervention to combat the epidemic (*2*). Médecins Sans Frontières (MSF) was one of the first nongovernmental relief organizations to respond to the epidemic and became the main organization supporting the MSPP in providing case management; more than half of all cholera patients nationwide received treatment in MSF-supported facilities (*3*,*4*).

The surveillance systems in place at the onset of the epidemic were unable to provide accurate and timely information (*5*); thus, on November 1, 2010, the MSPP launched a dedicated national cholera surveillance system based on daily collection of data about cholera cases and cholera-related deaths recorded in healthcare facilities across the country and of community cases and deaths reported by community members. Information about cholera-related deaths -in the community was collected through a variety of channels including reports from physicians, community health workers, and community leaders (*6*). In addition, in November 2010 an alert and response surveillance system was implemented to complement the national cholera surveillance system and to better monitor the spread of the epidemic and guide prevention and control activities. The alert and response surveillance system collected broad information about any cholera event requiring immediate response (*7*). By mid-April 2011 (end of the first wave of the cholera epidemic), 283,362 cases of cholera had been reported to the national cholera surveillance system, including 152,816 hospitalizations and 4,856 deaths (*6*). Although large, this number of deaths implies a small (≈1.1-fold) increase in the crude mortality rate for Haiti, where ≈90,000 deaths are expected to occur annually (*8*). According to the national cholera surveillance system data, by mid-January 2011, the case-fatality rate within healthcare facilities dropped to <1%, indicating improved cholera case management (*6*,*9*).

However, a rapid assessment of cholera-related deaths, conducted by active case finding in Artibonite Department in November 2010, estimated that 87% of deaths were not recorded in the hospital records (*10*). These findings raised the possibility that a substantial number of cases and deaths across the country were not reported during the first wave of the epidemic, a prospect supported by subsequent assertions that the existing surveillance systems at the onset of the epidemic were unable to fully capture the amount and type of data needed to monitor the rapid evolution of the epidemic (*6*,*11*). If true, this assertion would imply that the public health consequences of this epidemic were underestimated and would raise questions about ways to improve the implementation and accuracy of cholera surveillance during epidemics so that these vital data are rapidly available to help first responders implement the most effective public health interventions possible.

For this reason, MSF conducted 4 retrospective community-based surveys (1 in each of 4 locations where MSF intervened) to assess the extent of deaths during the first phase of the epidemic in Haiti (mid-October 2010 through mid-April 2011). We present the findings of these surveys and new estimates of the magnitude of the death toll and crude mortality rates for this first epidemic wave of cholera in Haiti.

## Methods

The study was conducted in collaboration with the MSPP after obtaining permission to conduct the survey. The study protocol was approved by the National Ethical Review Board of Haiti. Written consent was obtained from all study participants.

### Study Setting and Design

**Figure 1:**
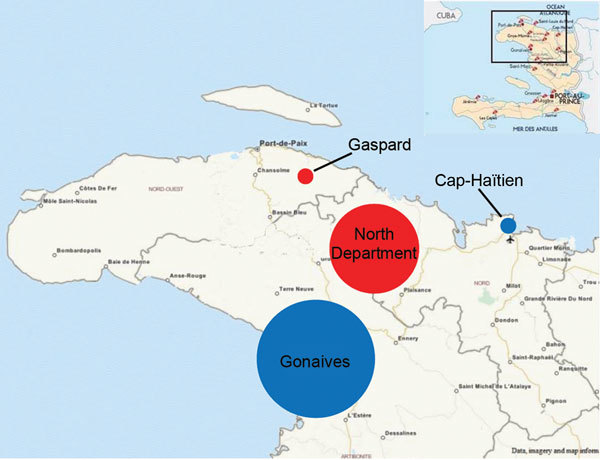
Study sites used to determine mortality rates during cholera epidemic, Haiti, 2010–2011: entire town of Gonaives, urban slum in Cap-Haïtien, rural communal sections in North Department, and communal section of Gaspard. Red circles, rural sites; blue circles, urban sites. Circle size is proportional to the estimated population of each site.

Of the 4 survey sites (Gonaives, Cap-Haïtien, North Department, and Gaspard and Zabricots), 2 were urban and 2 were remote rural areas (Figure 1). In Gonaives, the main town of Artibonite Department, the survey covered the entire town. In Cap-Haïtien, the capital of North Department, the study was conducted in a densely populated slum. In Gaspard and Zabricots, the survey was conducted in a small, hilly section where poor road quality made road access difficult. The North Department rural site combined remote, isolated areas with areas of better access. These 4 sites were selected because of the large number of cases reported from them to the national cholera surveillance system during the first wave of the epidemic and because MSF had implemented a large intervention at each of them. These settings also represented diverse contexts (urban vs. rural, high vs. low population density, good vs. poor access to healthcare) where cholera could have evolved in different ways.

We used a core generic protocol for the 4 sites and then adapted the sampling approach to the different settings. At Gonaives and North Department, we conducted a 2-stage, household-based cluster survey. At the first stage of sampling, clusters were allocated to communal sections (administrative subdivisions of the source population) proportionally to their selected population size. At the second stage, we randomly selected the first household of each cluster through spatial sampling by using the R statistical package (*12*). The starting household of each cluster in the field was identified by use of a global positioning system. We then selected subsequent households by proximity, until the cluster was complete. At Cap-Haïtien and at Gaspard and Zabricots, every household was surveyed because of the small total populations (exhaustive surveys).

The cluster-based surveys were conducted during March 29–April 7, 2011, in Gonaives and during April 23–May 13, 2011, in North Department. The exhaustive surveys were conducted during April 11–29, 2011, in Gaspard and Zabricots and during April 1–14, 2011, in Cap-Haïtien.

### Sample Size

The study design called for sampling 16,000 persons at each site. This sample size was sufficient to estimate a crude mortality rate of 18 deaths/1,000 person-years (95% CI 14–22), which represents twice the expected crude mortality rate for 2010 in Haiti (9 deaths/1,000 person-years) by the United Nations World Population Prospects (*8*) (Technical Appendix). The study involved a recall period of 170 days and a hypothetical design effect (loss of variance because of intracluster homogeneity) of 2.

### Data Collection

The same questionnaire was used at each of the 4 sites. Survey teams recorded data about deaths that occurred during predefined recall periods starting on October 17, 2010 (epidemiologic week 42 in 2010) and ending on the date of the interview in 2011 (no later than May 13, 2011). At all sites, death recall periods included the first (main) wave of the cholera epidemic in Haiti.

Trained interviewers administered the questionnaires to the head of the household or the most senior adult responsible for the household present at the time of the interview. The questionnaire asked about deaths in the household that occurred within the recall period. For each death, we reported the date and the age of the deceased (in years) and coded the reported cause of death. In addition, we asked about episodes of watery diarrhea during the recall period and the outcome of these episodes.

To facilitate recall of the survey period, we used a calendar of locally important events. We also asked about family members who were absent and about persons who were visiting the household; we excluded from the denominator periods when household members were absent for >2 weeks and visitors stayed for <1 month. We asked all respondents for the age and sex of living household members.

### Estimated Deaths

The crude mortality rate and watery diarrhea–specific mortality rate were each expressed as deaths per 1,000 person-years; we used as the denominator each person’s time at risk during the recall period. To estimate the diarrhea-specific mortality rate, we counted those for whom death was the reported outcome of a watery diarrhea episode.

We also calculated mortality rates per epidemiologic week (*13*) by dividing deaths that occurred in these periods by the total person-time spent by the surveyed population in each week. The number of days at risk for each person was determined as the difference between his/her date of entry into the household (birth, moving in, or October 17, 2010) and date of exit (death, moving out, or interview date).

The expected number of deaths in the absence of an epidemic was computed by using as a baseline the expected mortality rate for Haiti in 2010 provided by United Nations World Population Prospects (*8*), which was based on a combination of nationally representative household surveys, census reports, and death registries (*8*,*14*). The expected number of deaths was obtained by multiplying the expected mortality rate by the estimated person-years lived in the study areas. The number of excess deaths was calculated by subtracting the expected number of deaths in the absence of an epidemic from the estimated number of deaths during the study period.

### Statistical Analyses

Data were entered by using EpiData version 3.0 (EpiData Association, Odense, Denmark) and analyzed with Stata 10 software (StataCorp LP, College Station, TX, USA). Crude mortality rate point estimates were obtained by using a Poisson regression model; the design effect was estimated by using the STATA command “svy” to obtain 95% CIs.

## Results

For the cluster-based surveys in Gonaives and North Department, we randomly selected 105 and 138 clusters from which we included, respectively, 3,201 and 3,187 households. The total population surveyed and the household size was similar for all 4 sites (i.e., for the cluster-based and the exhaustive surveys, varying from 5.3 members in North Department to 6.2 in Gaspard and Zabricots). Median age varied from 19 years in Gaspard and Zabricots to 21 in North Department and Cap-Haïtien (Table 1).

**Table 1:** Sites and participants in study of mortality rates during cholera epidemic, Haiti, 2010–2011

Variable	Study site			
	Gonaives	Cap-Haïtien	North Department	Gaspard
Estimated population	228,725	16,000	173,904	17,000
No. clusters	105	Not applicable	138	Not applicable
No. households sampled	3,201	2,682	3,187	3,379
No. survey participants present on survey date	18,363	14,694	16,900	20,946
Average recall period, d	162	170	195	174
Median age (interquartile range), y	20 (11–30)	21 (12–32)	21 (11–40)	19 (9–36)
No. (%) children younger <5 y of age	1,921 (10.5)	1,482 (10.4)	1,690 (10.0)	2,574 (12.3)
Male-to-female ratio	0.84	0.87	0.91	0.99
Average household size (SD)	5.7 (2.5)	5.5 (3.1)	5.3 (2.3)	6.2 (2.8)
No. births	155	106	110	309

A total of 983 deaths were reported from the 4 sites (Table 2), corresponding to crude mortality rates (deaths/1,000 person-years) ranging from 19.1 in Gonaives to 35.4 in Gaspard and Zabricots. The most frequently reported cause of death was diarrhea. The second most frequently reported cause was respiratory tract infection.

**Table 2:** Crude and diarrhea-specific mortality rates during cholera epidemic, Haiti, 2010–2011

Variable	Study site			
	Gonaives	Cap-Haïtien	North Department	Gaspard
Study population	18,363	14,694	16,900	20,946
Person-years	8,121	6,230	9,027	10,004
No. deaths	159	194	275	355
Crude mortality rate (95% CI)*	19.1 (14.9–24.4)	28.4	30.2 (23.5–38.8)	35.4
No. diarrhea-related deaths	105	166	224	277
Diarrhea-specific mortality rate (95% CI)*	12.4 (8.9–17.2)	24.3	24.5 (18.5–32.6)	27.7

*No. deaths per 1,000 person-years, calculated by using Poisson regression taking into account the survey design.

Overall, 1,800 deaths were expected during the study period (average recall period 176 days) in the target population (438,505 persons), but we estimated that 5,296 deaths occurred. The difference between these numbers (i.e., 3,406 deaths) represents the excess deaths and corresponds to a 2.9-fold overall increase (ranging from a 2-fold increase in Gonaives to a 4-fold increase in Gaspard and Zabricots) for the 4.4% of the Haiti population covered by these surveys. Overall, we estimated that 3,999 diarrhea-related deaths occurred in the study population (Table 3).

**Table 3:** Excess deaths during cholera epidemic, Haiti, 2010–2011

Variable	Study site			
	Gonaives	Cap-Haïtien	North Department	Gaspard
Population	228,725	14,931	173,903	20,946
Person-years	101,167	6,936	93,026	10,028
Expected total no. deaths*	905	62	833	90
Estimated total no. deaths (95% CI)†	1,933 (1,512–2,472)	197	2,810 (2,186–3,612)	355
Risk ratio	2.1	3.2	3.4	4.0
No. excess deaths (95% CI)	1,028 (606–1,567)	132	1,978 (1,354–2,780)	265
Estimated no. diarrhea-related deaths (95% CI)†	1,254 (900 – 1,740)	169	2,279 (1,721–3,033)	278

*Calculated by multiplying the expected baseline mortality rate in Haiti for the year 2010 in the absence of an epidemic (8.95 deaths/1,000 person-years) by the population (person-years). †Estimated according to answers to the questionnaire.

**Figure 2:**
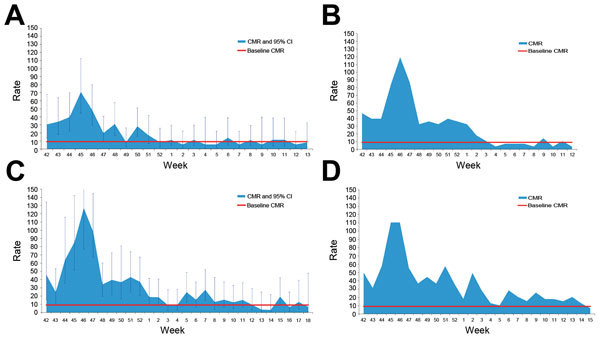
Crude mortality rate (CMR; no. deaths/1,000 person-years), by week, at study sites used to determine mortality rates during cholera epidemic, Haiti, 2010–2011. A) Gonaives; B) Cap-Haïtien; C) North Department; D) Gaspard. Red line indicates the expected crude mortality rate for Haiti in 2010 in the absence of an epidemic. Error bars indicate 95% CIs.

The excess deaths were not distributed equally over time. The highest number of deaths occurred in 2010 during epidemiologic weeks 44–52 (October 17, 2010–1 January 1, 2011) in the 4 sites, reaching 127 deaths per 1,000 person-years in North Department—a 14-fold increase compared with baseline mortality rates in Haiti (Figure 2). After January 1, 2011, the crude mortality rate started to decrease, and by the end of the recall period, the rate returned to the baseline crude mortality rate expected for Haiti (Figure 2).

## Discussion

From October 2010 through April 2011 at the 4 study sites in Haiti (which covered 4.4% of the Haiti population), the crude mortality rate increased by an estimated 2.9-fold (2.1–4.0-fold across sites) compared with baseline data, which corresponds with 3,406 excess deaths. However, the official number of cholera deaths reported for the entire country during the study period was 4,856 (*6*,*7*), which would represent an ≈1.1 fold increase in the crude mortality rate. In Gonaives, where a direct comparison between the number of deaths calculated in our study and the number of cholera deaths reported by national cholera surveillance was possible, we estimated 1,254 watery diarrhea–related deaths and 1,028 excess deaths, whereas the national cholera surveillance system reported only 132 cholera deaths during the same period (*6*,*7*). Considering the high attack rates reported throughout most of the country during this period (*6*,*7*), our results suggest a larger effect of the epidemic on the mortality rates than previously reported in Haiti.

Most of the deaths we recorded occurred during the first weeks of the epidemic and were attributed by survey respondents to watery diarrhea. We found that during January 2011, the crude mortality rate in the 4 study areas decreased to baseline rates (i.e., similar to estimates for 2010 that do not account for the epidemic), indicating that the largest effect on mortality rates occurred during the first 6 weeks of the epidemic.

Several limitations should be considered when interpreting our findings. First, because the study assessed deaths retrospectively, recall bias might have occurred. Although recall bias associated with ascertaining death events per se is unlikely, considering the proximity between the events and the surveys, recall bias might have influenced the accuracy of reported dates, cause of death, or both. In particular, overreporting of diarrhea as cause of death is possible, considering the strong psychological effects of the cholera epidemic on the Haiti population during this period. We tried to minimize the effect of this possible bias in our calculation of the diarrhea-specific mortality rate by including in this calculation death reported as an outcome of a clearly identified diarrhea episode, rather than death with diarrhea cited as a cause of death. However, this effort may have been insufficient to entirely correct this bias.

Another limitation is that, despite efforts to be exhaustive in Gaspard and Zabricots and in Cap-Haïtien, 12% of the estimated population of the Cap-Haïtien slum was not included in the survey. This lack of coverage might be explained by an inaccurate estimate of the official population size, by missed households, or both. If the excluded population differed from the study population in terms of deaths, the Cap-Haïtien estimates would be biased.

In the absence of exceptional events, mortality rates generally follow stable trends (Technical Appendix). The baseline mortality rates that we used for calculation of excess deaths in Haiti are internationally accepted as valid indicators of the rate of death occurrences (*8*,*14*). Variations in completeness and accuracy of the data sources might bias these baseline estimates. As an example, for Haiti during 2010–2015, the United Nations World Population Prospects provides a low variant of 8.5 deaths and a high variant of 8.7 deaths per 1,000 person-years. To assess the possible bias associated with the baseline mortality rate assumption, we conducted a sensitivity analysis (online Technical Appendix), which showed a low effect of these variations. Seasonal variation of morality rate could also partially explain the excess deaths; however, the strong correlation between the crude mortality rates and the epidemic curve suggest a true association between the excess deaths and the peak of the epidemic. In addition, the high proportion of deaths attributed to diarrhea relative to the expected proportion (≈85% vs. 5%–16%; [*15**–**18*]) is consistent with the existence of high numbers of deaths from cholera.

As expected, the overall crude mortality rates varied by region; however, the only baseline available was a national average, not regionally specific crude mortality rates for Haiti. The site-specific estimates of excess deaths may be less accurate because the local baseline crude mortality rates may be higher or lower than the national average. Because our surveys included a range of contexts (e.g., urban, rural, good and poor access to healthcare), pooled comparisons are probably largely representative of the excess deaths caused by the cholera epidemic in areas with high incidence of cholera. However, cholera incidence rates at the study sites may have been higher than those in other regions because these sites were selected for their large number of reported cases.

Before the establishment of the national cholera surveillance system and the alert and response surveillance system, health surveillance relied on 2 syndrome-based disease-surveillance systems that were implemented after an earthquake occurred near the capital of Port-au-Prince in January 2010 (*19*,*20*). However, these systems were insufficient for handling the amount and type of data needed to monitor the evolution of the cholera epidemic (*6*). Because surveillance is a cornerstone in any epidemic response intervention providing essential information to guide prevention and control strategies, a comprehensive cholera surveillance system was required. However, if the estimates presented here are correct, then many deaths in Haiti were never counted in the official statistics during the first wave of the cholera epidemic despite the commendable effort to promptly implement a national cholera surveillance system.

Prior cholera epidemics have shown the limits of traditional response strategies for reducing the spread of the epidemics (*21*); but for many epidemics, the response interventions have been considered successful to limit the number of deaths (*22*). In Haiti, the national cholera surveillance system showed similar trends; however, our study results suggest that that the cholera-associated mortality rates have been substantially underestimated. These results imply that the outbreak response strategy was insufficient for avoiding a high number of deaths in the first weeks of the epidemic despite the enormous effort made by the MSPP, the World Health Organization, MSF, and other agencies to improve access to appropriate treatment for cholera patients.

The high mortality rate documented in our study during the first weeks of the epidemic might be associated with different factors. The healthcare system in Haiti had been severely strained by the 2010 earthquake (*23*). Although none of the areas included in our survey were directly affected by the earthquake, the national health services were still rebuilding when the cholera epidemic began (*6*,*19*,*24*). In addition, cholera was an unknown disease for the Haiti population, including medical staff who were not accustomed to treating the rapid clinical evolution of dehydration associated with severe cholera. Likewise, members of the population were unaware of how to prevent cholera and the value of promptly seeking care at the onset of signs and symptoms.

Because most cholera-associated deaths occur on the first day of sickness (*10*,*25*), early access to care is critical for improving survival rates. Thus, among the crucial steps for reducing cholera-associated deaths are decentralizing medical care and creating public awareness about cholera and where to seek care. However, decentralization of healthcare structures in Haiti was and remains difficult in very remote areas such as some villages in North Department that require a 10-hour walk to get to the nearest healthcare facility. This link between healthcare access and cholera deaths is consistent with our observation of large differences in mortality rates across the 4 study sites; for example, the mortality rate for the most remote area of Gaspard was almost twice as high as that for urban Gonaives (35.4 vs. 19.1 deaths/1,000 person-years, respectively). Although further investigations are required to fully interpret these figures, distance and ease of accessing care are most likely contributing factors (*26*). Innovation is needed to improve the promptness of establishing access to healthcare, especially in remote areas. Involving communities in preparedness plans for epidemics might be a promising approach (*27*,*28*). New tools for preventing cholera should be considered, such as innovative water treatment systems, new sanitation solutions, and vaccines. At the onset of the cholera epidemic in Haiti, a limited number of vaccine doses were available, but they were not used in the control strategy. Since 2010, vaccine supply and use have increased worldwide, including in Haiti, and vaccination is becoming an additional tool that should be considered for outbreak prevention and control (*29*,*30*), especially where good access to healthcare cannot be made rapidly available or guaranteed over time. We also consider essential the provision of clear guidance on ways to improve current epidemic response plans from the World Health Organization and the Global Task Force for Cholera Control (*31*).

Our study findings offer some implications for surveillance and the response strategy in future epidemics. The results suggest that relying on surveillance based primarily in healthcare facilities provides a biased picture of an epidemic and underestimates illness and death from the disease, especially if the surveillance system has weaknesses and requires adaptation during the first phase of the epidemic. This limitation has been documented in Zimbabwe, where community-based studies showed underreporting of cholera-related deaths (*32*). Rigorous assessments at the community level, including surveys and community-based surveillance, are essential for accurately estimating the true extent of cholera illness, death, and socioeconomic cost. In the absence of better estimates, cholera will remain a neglected problem for less-developed countries if the attention, innovation, and funding allocated are insufficient for improving the current control efforts.

In conclusion, our study findings suggest that the mortality rate during the cholera epidemic in Haiti was larger than that reported in the official statistics (*6*). Cholera epidemics are primarily surveyed through information collected in healthcare facilities; however, this type of surveillance might not be enough to describe the true extent of cholera, especially in places where the healthcare systems are weak. Community-based systems should be reinforced to complement healthcare facility–based systems. Affected communities should also be more involved in preparedness and response strategies because they can effect timely provision of oral rehydration therapy, promote prompt seeking of healthcare, and integrate new preventive tools into local practices. Clear leadership and international consensus are required to improve current epidemic response strategies, which should ultimately stop cholera from causing a large and avoidable number of deaths.

**Technical Appendix.** Estimated excess deaths in study population, calculated by using low, medium, and high variants, Haiti, 2010; observed and expected crude mortality rate estimates for Haiti, 1970–2020.
