# Supplementary material for: Mortality Rates during Cholera Epidemic, Haiti, 2010–2011
Source: Emerg Infect Dis. 2016 Mar;22(3):410–6. doi: 10.3201/eid2203.141970 (PMC4766911; doi:10.3201/eid2203.141970)
Supplement: Supplementary file 1 — Technical Appendix. Estimated excess deaths in study population, calculated by using low, medium, and high variants, Haiti, 2010; observed and expected crude mortality rate estimates for Haiti, 1970–2020. [file 14-1970-Techapp-s1.pdf]

Article DOI: http://dx.doi.org/10.3201/eid2203.141970

Mortality Rates during Cholera Epidemic, Haiti, 2010–2011

Technical Appendix

Technical Appendix Table. Estimated excess deaths in the study population using the low, medium, and high variant for the year 2010 in Haiti from the United Nations World Population Prospects (http://data.un.org/Data.aspx?d=PopDiv&f=variableID:65)

Study area	Gonaives	Cap-Haïtien	North Department	Gaspard	Total
Population	227,715	14,931	181,118	20,946	444,710
Expected number of deaths - low variant	892	61	858	89	1,900
Expected number of deaths - medium variant	902	62	867	90	1,921
Expected number of deaths - high variant	912	63	877	91	1,943
Estimated number of deaths	1,890	218	2,926	355	5,389
Excess deaths - low variant	998	157	2,068	266	3,489
Excess deaths - medium variant	988	156	2,058	265	3,468
Excess deaths - high variant	978	155	2,048	264	3,446

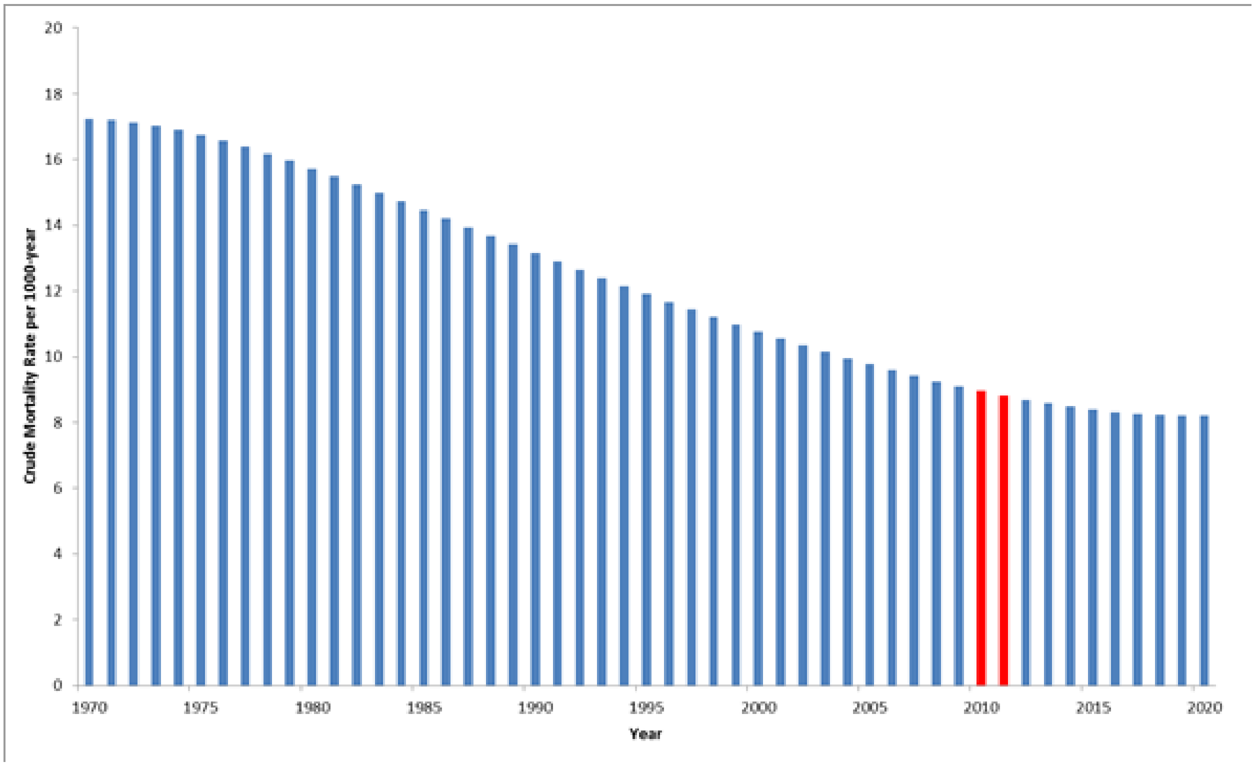

Technical Appendix Figure. Observed and expected crude mortality rate estimates for Haiti in the period 1970 to 2020 from the United Nations World Population Prospects (http://data.un.org/Data.aspx?d=PopDiv&f=variableID:65). The crude mortality rate has decreased from 17.2 deaths per 1000 person-year in 1970 to less than 10.0 deaths per 1000 person-year in 2013; the

expected crude mortality rate was 9.0 deaths per 1000 person-year for 2010 and 8.8 deaths per 1000 person-year for 2011.
